# Proteinase Activated Receptor 4 in the Jejunum of Healthy Horses and of Horses With Epiploic Hernia

**DOI:** 10.3389/fvets.2020.00158

**Published:** 2020-03-31

**Authors:** Carlotta Lambertini, Cristiano Bombardi, Augusta Zannoni, Chiara Bernardini, Francesco Dondi, Maria Morini, Riccardo Rinnovati, Alessandro Spadari, Noemi Romagnoli

**Affiliations:** Department of Veterinary Medical Sciences, University of Bologna, Ozzano Dell'Emilia, Italy

**Keywords:** epiploic foramen hernia, equine, jejunum, mast cells, proteinase-activated receptor 4

## Abstract

Proteinase activated receptor 4 (PAR_4_) in the gastrointestinal tract is involved in the regulation of inflammation and pain pathways. The aim of the present study was to evaluate the distribution and expression of PAR_4_ in the jejunum of healthy horses and in the pathologic tracts from horses undergoing surgery for herniation of the small intestine through the epiploic foramen. Eight healthy horses (Group H) and eight horses with epiploic hernia (Group EH) were included; the jejunum samples were collected at the slaughter or intraoperatively after enterectomy, respectively. To evaluate PAR_4_ expression in sections of the jejunum, immunofluorescence, western blot and quantitative polymerase chain reaction (qRT-PCR) were performed. Immunohistochemistry of PAR_4_ in the jejunum of the healthy horses showed that receptors are predominantly expressed in the immune cell population scattered throughout the lamina propria of the mucosa and in the submucosa. Quantitative PCR data demonstrated that PAR_4_ mRNA was detectable in all of the samples analyzed without any difference between the H and the EH groups, however the PAR_4_ protein level was significantly lower in the jejunums of the EH horses. In the Group EH horses, PAR_4_ immunoreactivity was mainly expressed in the mast cells and was extensively distributed in the sierosa. In the lamina propria of mucosa of Group EH, leukocytes were less abundant than in Group H. In this study, the distribution and expression of PAR_4_ in the jejunums of the healthy horses and in those with spontaneous occurring epiploic hernia was demonstrated.

## Introduction

In horses, herniation of the small intestine through the epiploic foramen (epiploic hernia-EH) is a common cause of strangulation of the small intestine and has been reported from 3.5 to 5% of horses with colic undergoing surgery (4, 5). More frequently, compression within the foramen or anatomical displacement cause vein occlusion with subsequent tissue congestion, oedema, hemorrhagic infarction, and inflammation (6). Moreover, when artery compression develops, ischemic lesions also appear (7). Tissue necrosis can advance up to one meter involving the afferent and efferent intestinal loops not involved in the hernia (1).The surgical treatment of an EH consists of reducing the herniation and resection of the abnormal intestinal tract (7). However, reperfusion of the ischemic intestine might results in reperfusion injury characterized by the aggravation of the aforementioned lesions (2, 8).

Several studies in humans and in animal models have investigated the role of proteinase activated receptors (PARs) in the pathophysiology of intestinal diseases (9–12). Proteinase activated receptors constitute a receptor subfamily belonging to the largest group of G-protein coupled receptors (13–16). In humans, PARs 1 to 4 have been identified throughout the gastrointestinal tract where they are exposed to high levels of proteinases, digestive proteinases, and those produced by enteric pathogens, and are therefore relevant in the regulation of several mechanisms (15, 17–19). In addition the expression of proteases increases significantly during inflammatory processes (17). The proteases interact with PARs by cleaving the extracellular N-terminus portion; the tethered ligand that in this way is exposed binds to a second extracellular ring of the receptor thereby activating the receptor itself (9). Proteinase activated receptor 4 is activated by trypsin, cathepsin G and activated factor X and is considered particularly involved in the regulation of inflammation, nociception, sensitization, and pain pathways (20–23). In humans, authors are investigating the role of PAR_2_ and PAR_4_ in the pathogenesis of irritable bowel syndrome and in the modulation of hypersensitivity associated with persistent tissue inflammation (10, 12). The expression and distribution of PAR_4_ in the equine small intestine has not yet been investigated.

The aim of the present study was to evaluate the distribution and expression of PAR_4_ in the jejunums of healthy horses and in the ischemic tracts collected from horses undergoing surgery for colic of the small intestine. Therefore, horses with herniation of the small intestine through the epiploic foramen were considered as model of inflammation and strangulating obstruction of the small intestine.

To evaluate PAR_4_ expression in sections of the jejunum, immunofluorescence, and gene and protein expression studies were carried out.

## Materials and Methods

### Animals

The study was approved by the Ethical Scientific Committee for experimental animals of the University of Bologna (Prot. n 15-IX/, 08/05/2012). Eight healthy horses (group H) and eight horses with EH (group EH) were included in the study.

In Group H, eight adult food-producing Standardbred horses sent to slaughter were included. These horses were considered healthy based on history, and physical and clinicopathological examination, and on the basis of gross and histological evaluations of the jejunum. There were five females and three geldings of different breeds; their median age was 10.5 years (range 2–20). In Group EH, eight adult sport Standarbred horses (three females and five geldings) admitted to the Veterinary teaching hospital (VTH) of the Department of Veterinary Medical Sciences for colic syndrome and in which an exploratory laparotomy confirmed a clinical diagnosis of herniation of the small intestine through the epiploic foramen were included. All patients underwent an enterectomy of the jejunum followed by anastomoses within 2-18 h from admission. At the end of the surgery, horses with colic were followed by the VTH and discharged within 7–10 days from admission.

### Blood Sampling and Analysis

In the Group H horses, blood samples were collected from the jugular vein at exsanguination. In the Group EH horses, blood samples were collected within 30 min from admission from the jugular vein by venipuncture. Blood samples were collected in K3EDTA, citrate and clot activator. In detail, a complete blood count was carried out including: hematocrit value, hemoglobin concentration, erythrocyte indices, platelet count, and white blood cells and differential white blood cells counts. The chemical profile included aspartate transaminase, lactate dehydrogenase, creatine kinase, creatinine, urea, glucose, lactate, total bilirubin, γ-glutamyl transferase, total protein, total calcium, phosphorus, sodium, potassium, and chloride. The coagulation profile included D-dimers and antithrombin. The acute-phase protein profile included serum amyloid A, haptoglobin, ferritin, fibrinogen, total iron, transferrin (such as total iron binding capacity), and albumin. The samples collected were processed and analyzed within one hour or stored at −80°C. The blood analysis were carried out using an automated hematology system (ADVIA 2120, Siemens Healthcare Diagnostics, Tarrytown NY, USA) and an automated chemical analyzer (AU 400, Olympus/Beckman Coulter, Brea CA, USA).

### Sampling and Morphological Evaluations

In the group H horses, samples of the jejunum were collected at the evisceration phase of the slaughter. At the time of collection, samples were examined internally and externally to exclude gross intestinal lesions. In the group EH horses, the abnormal tract of the jejunum was resected intraoperatively and a jejuno-jejunal or jejuno-ileal anastomosis was performed by an experienced surgeon. The ischemic intestinal tract removed was used for research purposes with the informed consent of the owners. All the samples collected for the analysis were obtained in the center of the abnormal removed intestine.

After collection, a part of the jejunum was dissected and washed with phosphate buffered saline (PBS) (Gibco-Invitrogen, Paisley, UK). The intestinal mucosa was then scraped with two glass slides; the samples were frozen in liquid nitrogen and stored at −80°C until RNA and protein extraction were carried out. In addition, full thickness bioptic samples from the jejunum were carried out, rinsed in PBS and then immediately stored in 10% neutral buffered formalin and 4% paraformaldehyde.

Histological evaluations were carried out on samples fixed in 10% neutral buffered formalin, embedded in paraffin within 24–48 h, cut into 5 μm thick sections and stained with hematoxylin and eosin. For group H, the histological samples were also evaluated according to an inflammation score using a semiquantitative estimation of the inflammatory cells in the mucosa and submucosa, as described by Packer et al. (24). Only those horses in which the intestinal specimens were within the normal range were included in the study. In addition, for samples of both groups, tissue viability was determined based on the morphological changes present in the tissue sections using light microscopy and was based on a scoring system previously described by Van Hoogmoed et al. (25).

### Immunohistochemistry Experiments

In both groups, samples for immunohistochemical evaluation were prepared as previously described (26) to obtain 14–15 μm thick cryostat serial longitudinal sections, which were mounted on polylysine-coated slides and stored at −80°C until immunohistochemical evaluations were carried out. The sections were then rehydrated three times with PBS (0.02 M, pH 7.4) before beginning the immunostaining. To block non-specific binding, the sections were incubated in a solution containing 10% normal donkey serum (Colorado Serum Co, Denver, CO, USA) and 0.5% Triton X-100 (Merck, Darmstadt, Germany) in PBS for an hour at room temperature in a humid chamber. An hour later the sections were incubated in primary antibody goat anti-PAR4 (1:100, Santa Cruz Biotechnology Cat# sc-8464, RRID:AB_653835) diluted in antibody diluent (1.8% NaCl in 0.01 M sodium phosphate buffer containing 0.1% sodium azide), 1% normal donkey serum and 0.5% Triton for 24 h at room temperature in an humid chamber. The day after, the sections were washed with PBS three times every 10 min and incubated in a solution containing a secondary antibody donkey anti-goat (1:400, Alexa Fluor 594, Thermo Fisher Scientific, Waltham, MA, USA, Cat# A-11058, RRID:AB_2534105), 1% normal donkey serum, 0.5% Triton diluted in PBS in humid chamber. Finally, the slides were washed three times with PBS and were then cover slipped with buffered glycerol (pH 8.6).

Thereafter double immunohistochemistry evaluations were performed adding different primary and secondary antibodies to the solution previously described. The sections used for mast cells assessments were incubated for 24 h at room temperature with a primary antibody solution containing the antibody rabbit anti-tryptase (1:80 Cloud clone corp, Huston, TX, USA, PAB07Ca01). These sections were then washed in PBS (three times for ten minutes each) and then incubated with a secondary antibody solution containing the antibody donkey anti-rabbit (1:200, Jackson ImmunoResearch Labs Inc., West Grove, PA, USA; Cat# 711-095-152, RRID:AB_2315776). The slides were then washed in PBS (three times for ten minutes each) and finally coverslipped. The section used for lymphocytes T and B assessment were incubated for 24 h at room temperature with a primary antibody solution containing the antibody mouse anti-CD3 (1:100; Sigma-Aldrich, Saint Luis, MO, USA, Cat# C7930, RRID:AB_259074) or mouse anti-CD79 (1:100 Abcam, Cambridge, UK, Cat# ab3121, RRID:AB_303528) respectively. The sections were then washed in PBS (three times for ten minutes each) and then incubated with a secondary antibody solution containing the antibody donkey anti-mouse (1:200, Jackson ImmunoResearch Labs Inc., West Grove, PA, USA, Cat# 715-095-150, RRID:AB_2340792). The slides were then washed in PBS (three times for ten minutes each) and finally cover slipped.

An average of 10 sections were evaluated for each horse. The immunofluorescence sections were observed using a Nikon H550L (Nikon instrument, Japan) equipped with a TRITC filter for Alexa 594 (EX 540/25; DM 565; BA 605-655) and a FITC filter for Alexa 488 (Ex 465–495; DM 505; BA 515–555). The images were recorded using a Nikon Qi1Mc photocamera (Nikon instruments, Japan) and Nikon Elements Version 4.10 software; the images were finally corrected for contrast and brightness in order to reproduce the appearance observed through the microscope, using Adobe Photoshop CS3 Extended 10.0 software (Adobe systems, San Jose, CA, USA).

### RNA Isolation and Quantitative Real Time PCR (qRT-PCR) for PAR_4_

The RNA extraction was performed on samples collected from each horse from the group H (*n* = 8) and the group EH (*n* = 8); RNA extraction and qPCR analysis were essentially carried out as reported by a previous study (26). Briefly, samples of scraped jejunum mucosa were pulverized using a mortar and pestle and liquid nitrogen; 25 mg of each sample were then lysed in Tissue Lyser TL (50 Hz for 5 min, 2 beads diameter 2 mm, QIAGEN, Hilden, Germany). The total RNA extraction was carried out using the NucleoSpin RNA II (Macherey Nagel, Duren, Germany) instructions. The RNA was spectrophotometrically quantified (A260 nm), and its quality was determined by gel electrophoresis on 1% agarose. Subsequently, 1 μg of RNA was reverse-transcribed to cDNA using an iScript cDNA Synthesis Kit (Bio-Rad Laboratories Inc., Hercules, CA, USA) in a final volume of 20 μl. Real-time quantitative PCR was carried out using a CFX 96 Real Time System (Bio-RAD Laboratories Inc.) and iTaq Universal SYBR Green Supermix (Bio-RAD Laboratories Inc.). All samples were analyzed in duplicate (10 μl/well).

The PAR_4_ and reference genes (GAPDH; HPRT and β-Actin) primer sequences, expected PCR product lengths and the accession numbers in the National Center of Biotechnology Information (NCBI) database are shown in Table 1.

**Table 1:** The proteinase activated receptor 4 (PAR_4_) and references gene (GAPDH; HPRT; β-Actin) forward and reverse primer sequences, expected PCR product lengths and accession number (AN) in the NCBI (National Center of Biotechnology Information) database.

**Gene**	**Sequence (5^**′**^-3^**′**^)**	**PCR length (bp)**	**AN**	**References**
PAR-_4_	For: ATGCGATCCTGCTGCTGTG	112	XM_001499653	Present study
	Rev.: GCGTCACTGTCACCGTCC			
GAPDH	For.: TGGTGAAGGTCGGAGTAAAC	120	NM_001163856	Zannoni et al. (26)
	Rev.: TGTAGTTGAGGTCAATGAAGGG			
HPRT	For.: GCGTCGTGATTAGTGATGATGAAC	179	AY372182	Zannoni et al. (26)
	Rev.: ACAGAGTGCTACAATGTGATGGC			
β-Actin	For.: ATCGTGCGTGACATCAAGGA	169	AF035774.1	Zannoni et al. (26)
	Rev.: AGGAAGGAGGGCTGGAAGAG			

The expression level of the PAR_4_ gene was determined using the 2^−ΔΔCt^ method (27) in which ΔCt = (Ct _PAR4_-Ct _mean ref.genes_) and ΔΔCt = ΔCt _EH−group_-ΔCt _H−group_.

### PAR_4_ Western Blotting

One sample collected from each horse of the group H (*n* = 8) and of the group EH (*n* = 8) was used for the western blot analysis.

Fifty mg of pulverized jejunum mucosa were lysed with sodium dodecyl sulfate solution (Tris-HCl 50 mM pH 6.8; sodium dodecyl sulfate 2%; glycerol 5%; 500 μl) supplemented with a protease inhibitor cocktail (P8340, Sigma-Aldrich, Co, St. Louis, MO, USA) in Tissue Lyser TL (50 Hz for 2 min, 3 beads diameter 2 mm). The protein content of the samples was determined using a Protein Assay Kit (TP0300, Sigma-Aldrich, Co, St. Louis, MO, USA). Aliquots containing 20 μg of protein were separated using Bolt 4–12% bis-Tris Plus (Life Technologies Ltd, Paisley, UK) for 55 min at 165 V. The proteins were then electrophoretically transferred onto a nitrocellulose membrane using the Turbo Blot System (Bio-Rad Laboratories Inc., Hercules, CA, USA). The blots were washed in PBS, and protein transfer was checked using 0.2% Ponceau Red staining. Non-specific binding on nitrocellulose membranes was blocked with 5% milk in PBS-T20 (phosphate buffer saline-0.1% Tween-20) for 1 h at room temperature. The membranes were then incubated overnight at 4°C with a 1:200 dilution of primary antibody goat anti-PAR4 (sc-8464, Santa Cruz Biotechnology, Dallas, TX, USA) in Tris Buffered Saline-T20 (TBS-T20 20 mM Tris-HCl, pH 7.4, 500 mM NaCl, 0.1% T-20). After several washings with PBS-T20, the membranes were incubated with the secondary donkey anti-goat IgG-HRP antibody (sc-2020, Santa Cruz Biotechnology Cat# sc-2020, RRID:AB_631728, 1:7000 dilution in TBS-T20, 1 h at RT). The Western blots were developed using chemiluminescent substrate (Clarity ECL Western Blot Substrate, Bio-Rad Laboratories Inc., Hercules, CA, USA) according to the manufacturer's instructions. The intensity of the luminescent signal of the resultant bands was acquired using the Chemi Doc Instrument (Bio-Rad Laboratories Inc., Hercules, CA, USA) with LabImage Software (Bio-Rad Laboratories Inc, Hercules, CA, USA).

In order to normalize the PAR_4_ data on the reference protein, the membranes were washed for 5 min in water, then for 5 min in 0.2 M NaOH and then washed again in water and reprobed for the reference α-tubulin (1:500; cod.TU-01 Thermo Scientific Waltham, MA, USA). The relative PAR_4_ protein content was normalized on the mean value of the reference protein (α-tubulin) and was expressed as arbitrary units.

### Statistical Analysis

Descriptive statistic was used to evaluate the clinicopathological data, and the reference intervals of our laboratory were used for comparison. The PAR_4_ protein and mRNA in the jejunum were compared between the H and the EH groups using a student *t*-test. *p* < 0.05 was considered statistically significant. The protein and mRNA analyses were carried out using GraphPad Prism software (La Jolla, CA, USA).

## Results

### Clinicopathological and Histopathological Evaluation

In group H horses the clinicopathological results were within the normal reference range. In group EH horses an increase in Serum amyloid A (median 149 μg/mL; reference range: 1–10 μg/mL), creatinine (median 2.15 mg/dL; reference range: 0.90–1.50 mg/dL) and d-dimer (median 2.90 μg/mL; reference range: 0.20–0.48 μg/mL) concentrations were observed. These results have already been published in a previous paper (28). At histological evaluation of the samples collected from the horses in group H, the median score was 2 (range 0–3). Twenty four samples were collected from the horses in group EH; in all of them the median score was 12 (range 6–40). In terms of hemorrhage and edema score (25) in this latter group, 14 samples had lesions of mild severity, while 10 samples had extensive disruption of structural integrity.

In the samples from group H, the quantitative data regarding the inflammatory cell populations was within the normal range as previously defined by Packer et al. (24).

### Immunohistochemistry

**Figure 1:**
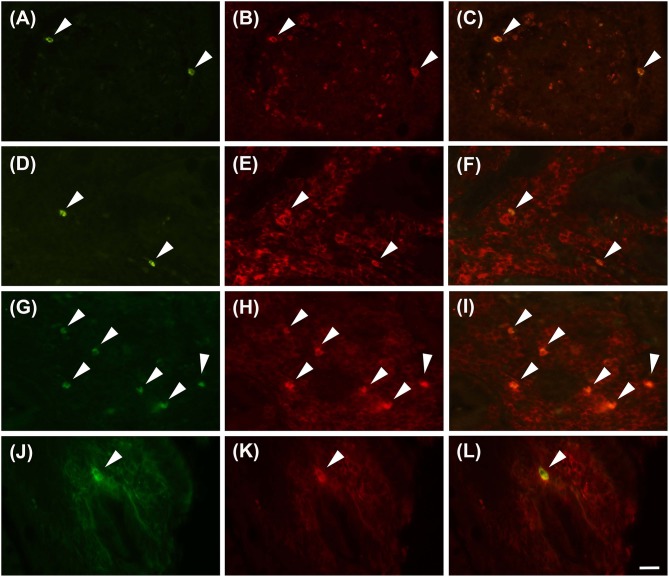
Lymphocytes immunoreactive for the PAR4 (PAR4-IR) in the lamina propria of the horse jejunum of group H **(A–F)** and group EH **(G–L)**. **(A–C)** and **(G–I)**: Anti-CD79 in green (LB), PAR4-IR in red (P) and co-localization of PAR4 and CD79 in B lymphocytes in yellow (LB+P). **(D–F)** and **(J–L)**: Anti-CD3 in green (LT), PAR4-IR in red (P) and co-localization of PAR4 and CD3 in T lymphocytes in yellow (LT+P). Arrowheads indicate double immunolabeled lymphocytes. Scale bar = 20 μm in L (applied to **A–L**).

**Figure 2:**
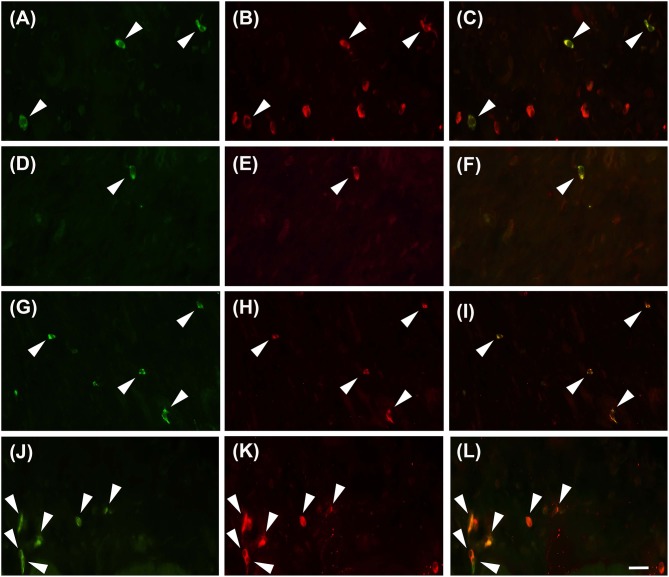
Mast cells immunoreactive for the PAR4 can be occasionally observed in the submucosa **(A–C, G–I)** and in the sierosa **(D–F, J–L)** in horse jejunum of group H **(A–F)** and group EH **(G–L)**. Tryptase in green (M), PAR4-IR in red (P) and co-localization of PAR4 with tryptase in yellow (M+P). Arrowheads indicate double immunolabeled mast cells. Scale bar = 30 μm in L (applied to **A–L**).

Proteinase activated receptor 4 immunohistochemistry in the jejunums of the healthy horses showed a weak receptor distribution, mainly in immune cell population, especially lymphocytes (Figure 1). These cells were predominant in the lamina propria of the mucosa and in the submucosa. In the submucosa, lymphocytes were observed only in a diffuse form. Proteinase activated receptor 4 immunoreactivity was not observed in the enterocytes and intestinal glands; in addition, the smooth muscle cells were negative with the exception of a few elements having weak immunoreactivity. In the superficial epithelium, lymphocytes with weak staining were observed. Some mast cells immunostained for tryptase were located in the submucosa (Figures 2A–C) and in the serosa (Figures 2D–F).

In group-EH horses, lymphocytes of the lamina propria of mucosa exhibited an intensity of immunofluorescence slightly lower than in group H. In the submuscosa and superficial epithelium some weakly-immunostained lymphocytes could be observed. In this group, PAR_4_ immunoreactivity was mainly expressed in the mast cells, immunostained for tryptase that were extensively distributed in the serosa (Figures 2G–L). The intensity of the immunofluorescence of the mast cells was similar in the two groups. Immunoreactivity was not observed in the submucosal and myenteric *plexi* either in Group H or in Group EH.

### Quantitative Real-time PCR for PAR_4_ in the Equine Jejunum

Quantitative PCR data demonstrated that PAR_4_ mRNA was detectable in all of the samples analyzed.

**Figure 3:**
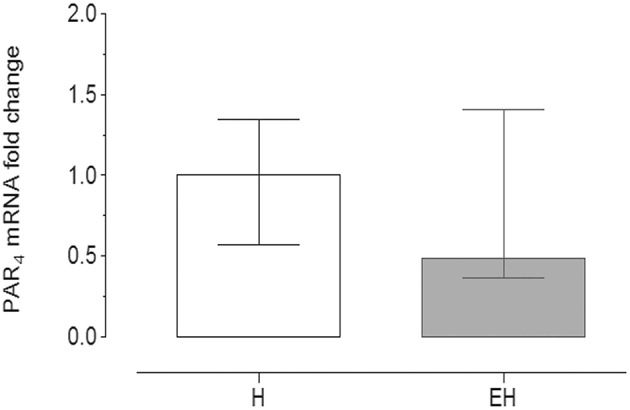
Quantitative Real time PCR for PAR_4_ in the equine jejunum tracts. The gene expression level of PAR_4_ in the EH group (*n* = 8) was calculated in relation to the samples isolated from the healthy group (H, *n* = 8), as fold of change (2 ^−ΔΔCt^ method in which ΔCt = Ct _PAR4_-Ct _mean ref.genes_ and ΔΔCt = ΔCt _EH−group_-ΔCt _H−group_). Error bars represent the range of relative expression of PAR_4_ in each group. No statistically significant differences in gene expression were observed (student *t*-test, *p* < 0.05).

No significant statistical difference of its expression in the jejunum tracts was observed between the groups EH (2^−ΔΔCt^ = 0.49) and H (2^−ΔΔCt^ = 1). The range of relative expression (lower and upper range), represented as error bars, was: 0.574–1.348 for H group and 0.365–1.41 for EH group (Figure 3).

### PAR_4_ Western Blotting in the Equine Jejunum

**Figure 4:**
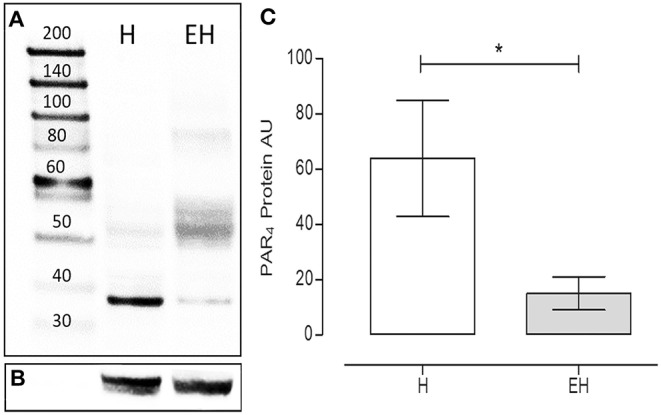
Proteinase activated receptor 4 protein expression in the jejunum intestinal tracts of equines with a diagnosis of epiploic hernia (EH, *n* = 8) and in healthy subjects (H, *n* = 8). Representative image of Western blots of the PAR_4_ protein **(A)** and the reference protein α-tubulin **(B)** in the intestinal tracts of equines. In lane 1, the molecular weight marker (kDa). Western blot analysis of PAR_4_ in the jejunum intestinal tracts isolated from the H and the EH groups **(C)**. A significant reduction in PAR_4_ expression was observed in the EH group) (student *t*-test, *p* < 0.05). AU = Arbitrary Unit. Data represent the mean ± SD of 8 biological replicates for each group. *represents a *p* < 0.05.

Under denaturing gel electrophoresis conditions (SDS–PAGE), western blots of the mucosa of the equine jejunum tracts showed a clear band of the expected molecular weight for PAR_4_ (~38 kDa) (Figures 4A,B). The PAR_4_ protein level was statistically lower in the jejunum tracts of the group EH (*p* < 0.05) (Figure 4C).

## Discussion

In the present study, the distribution and expression of PAR_4_ in the jejunum of healthy horses and in those with spontaneous occurring epiploic hernia has been demonstrated for the first time.

The expression and distribution of PAR_4_ has been investigated in humans and in mice (10, 12, 17, 19, 20, 22). In details, PAR_4_ mRNA expression has been identified in the small intestine and in the colon. In the colon the immunohistochemistry demonstrated the expression of PAR_4_ at the epithelium surface and crypts, in the submucosa and in the majority of mast cells (10, 12, 17). In the present study, immunoreactivity was not observed in the enterocytes and the submucosal cells, but mainly in the immune cell population identified as mast cells and B and T lymphocytes.

The expression of PAR_4_ in B lymphocytes has already been described in the human liver but authors did not find PAR_4_ mRNA in human circulating lymphocytes (29, 30).

The expression of PAR_4_ in mast cells is in accordance with previous studies in humans (10, 12). In particular, in human patients the inflammatory bowel disease resulted in a reduced expression of PAR_4_ in the colonic mast cells supporting its role in the modulation of visceral nociception (10, 12). Conversely, the results of the present study showed that the expression of PAR_4_ on mast cells in the jejunum did not change between healthy horses and those with epiploic hernia. However, we could not exclude that the lack of difference in the expression of PAR_4_ on these cells between the two groups is associated with the acute onset of the inflammatory process affecting the horses with epiploic hernia. The mast cells, in the peripheral tissues, modulate the nociceptive pathways, especially those of inflammatory origin. In fact, by means of mediators and cytokines release (tryptase and interleukin 1β) they activate other immune cells (neutrophils, macrophages and T cells) and amplify the inflammatory response (3, 31, 32). The mast cells in the intestinal tract seem to be particularly involved in the regulation of visceral hypersensitivity through PAR_4_ (3, 33, 34). In fact, *in-vitro* or *in-vivo* activation of PAR_4_ on mast cells resulted in a suppression of mRNA and protein levels of tryptase and 13 types of proinflammatory cytokines (3, 35). On the contrary the role of the PAR_4_ in lymphocytes is still to be clarified, however it does not seem to be involved in the modulation of nociception. In fact, Annaházi et al. (20) observed that in mice a functional deficiency of B and T cells did not affect the antinociceptive effect induced by the administration of a PAR_4_ activating peptide.

Based on these results, several authors are further investigating the involvement of PAR_4_ in visceral nociception and pain (3, 20–22). Augé and colleagues found that in mice, the intracolonic administration of a synthetic PAR_4_ activating peptide decreased the nociceptive response induced by visceral stimulation and inhibited the colonic hypersensitivity induced by the previous activation of the PAR_2_ (22). The same authors found also that PAR_4_-deficient mice showed enhanced pain behaviors (licking of abdomen, stretching and abdominal retractions) in response to colorectal stimuli as compared with wild type animals (22). In addition, the presence of PAR_4_ has been demonstrated in the dorsal root ganglia neurons where it reduces their excitability (36). These findings confirm the role of PAR_4_ not only in the modulation of nociception but also in the regulation of pain pathways.

A simultaneous activation of both PAR_2_ and PAR_4_ in the jejunum of horses with epiploic hernia and their involvement in the modulation of the inflammatory process associated with this pathological condition can't be excluded. In the equine bowel, the PAR_2_ expression has already been described either in healthy animals or in those with spontaneous occurring EH (26, 28). The results of these previous studies support an activation of the PAR_2_ during the ischemic process associated with intestinal herniation (28). Herein, the lower protein level for PAR_4_ found in the jejunums of horses with epiploic hernias, as compared with samples collected from healthy horses, supports the activation of PAR_4_ (37, 38). In fact, the activation of PARs by proteinases, is characterized by an irreversible proteolytic cleavage of the N-terminal domain of the receptor determining a conformational change of the receptors itself which is then internalized to reach intracellular effectors (37). After internalization PARs are degraded by lysosomes or recycled by endosomes (37). The degradation of the receptor can explain the decreased protein level observed in our study.

Proteinase activated receptors have been described also in the enteric nervous system where they are activated by proteinases, and they induce excitatory neuronal responses (18, 39). In the small intestine of the guinea pig, authors have found that submucosal plexus neurons express PAR_2_ and that a large proportion of myenteric neurons express PAR_1_ and PAR_2_ (40, 41). However, Mueller et al. (18) also found that stimulation with a PAR_4_ selective peptide induced a spike discharge in human and guinea pig submucosal neurons, even if the response was weaker and involved fewer neurons when compared with that obtained after the application of the PAR_1_ selective peptide. In horses, the submucosal plexus neurons and the myenteric plexus neurons expressed PAR_2_ (26, 28) but, in the present study, immunostaining for PAR_4_ was not observed in these cells. Therefore, based on the lack of expression of PAR_4_ in the enteric nervous system's neurons, it can be hypothesized that, in equine jejunums, this receptor is not involved in the regulation of neurotransmission in both healthy horses and in horses with epiploic hernia. Instead, PAR_2_ could be involved in the alteration of neurotransmission and intestinal motility during an inflammatory process as previously described in animal models (42). However, to date, there are no data concerning the role of PAR activation in the enteric nervous system in horses.

The increase in the SAA and plasmatic D-dimer and creatinine concentrations above the reference range are in accordance with previous studies in colic horses (43–46). In fact, the intestinal inflammatory process results in an increased concentration of acute phase protein like SAA (44, 47). In details, the increase of SAA concentration better correlates with the presence of inflammatory lesions compared with strangulating obstruction and in a previous study only the 21% of horses with pathologies involving the small intestine had an increase of SAA concentration above the reference range (44). In addition, disseminated intravascular coagulation is a complication related with acute gastrointestinal tract disease in horses. Increased plasma D-Dimer concentration and creatinine concentration have been previously reported in colic horses with coagulation disorders as marker of coagulopathies and subsequent organ damage respectively (43, 45, 48). However, clinical disseminated intravascular coagulation was not observed in the patients of the present study. The relationship between PAR_4_ expression and SAA was not evaluated. Further studies are needed to evaluate the application of the evaluation of PAR_4_ expression in association with SAA as a diagnostic and prognostic tool in horses with epiploic hernia.

In conclusion, these preliminary results support PAR_4_ expression in the equine jejunum and its potential activation during small intestinal herniation though the epiploic foramen. On the basis of previous studies, the local administration of the PAR_4_ activating peptide could be taken into consideration as a novel therapeutic approach for the treatment of inflammation and pain in horses with gastrointestinal disorders. In addition, as previously demonstrated in rodents, in equine species PAR_4_ activation might be involved in the modulation of visceral hypersensitivity associated with the PAR_2_ activation. However, the expression of PAR_4_ in the other intestinal tracts of the horse has still to be clarified and additional studies are needed to clearly define the role of PAR_4_ in the equine intestine.

## Data Availability Statement

The raw data supporting the conclusions of this article will be made available by the authors, without undue reservation, to any qualified researcher.

## Ethics Statement

The study was approved by the Ethical Scientific Committee for experimental animals of the University of Bologna (Prot. n 15-IX/, 08/05/2012). Written informed consent was obtained from the owners for the participation of their animals in this study.

## Author Contributions

CL, CBo, AZ, CBe, FD, and MM conducted data management, analysis, and interpretation of results. RR, AS, and NR performed samples collection. CL wrote the first draft of the manuscript. CBo and AZ wrote sections of the manuscript. NR and AZ contributed in conception and design of the study. All authors contributed to manuscript revision and approved the submitted version.

### Conflict of Interest

The authors declare that the research was conducted in the absence of any commercial or financial relationships that could be construed as a potential conflict of interest.

## Abbreviations

EH: Epiploic hernia
PAR: Proteinase activated receptor
PBS: Phosphate buffered saline.
